# A small molecule p53 activator attenuates Fanconi anemia leukemic stem cell proliferation

**DOI:** 10.1186/s13287-018-0882-5

**Published:** 2018-05-22

**Authors:** Wei Du, Xiaoli Li, Andrew F. Wilson, Qishen Pang

**Affiliations:** 1 Department of Pharmaceutical Sciences, West Virginia University School of Pharmacy, Morgantown, WV 26506 USA; 2 Division of Experimental Hematology and Cancer Biology, Cincinnati Children’s Hospital Medical Center, 3333 Burnet Avenue, Cincinnati, OH 45229 USA; 3 Department of Pediatrics, University of Cincinnati College of Medicine, Cincinnati, OH 45229 USA

**Keywords:** Fanconi anemia, Leukemic stem cells, Leukemogenesis, Nutlin-3, p53

## Abstract

Although p53 mutations are common in solid tumors, such mutations are found at a lower frequency in hematologic malignancies. In the genetic disorder Fanconi anemia (FA), p53 has been proposed as an important pathophysiological factor for two important hematologic hallmarks of the disease: bone marrow failure and leukemogenesis. Here we show that low levels of the p53 protein enhance the capacity of leukemic stem cells from FA patients to repopulate immunodeficient mice. Furthermore, boosting p53 protein levels with the use of the small molecule Nutlin-3 reduced leukemia burden in recipient mice. These results demonstrate that the level of p53 protein plays a crucial role in FA leukemogenesis.

Fanconi anemia (FA) is a genetic disorder caused by defects in at least 21 genes (*FANCA-V*) [1–6]. Patients with mutations in any of these genes develop a FA phenotype characterized by a variety of symptoms, including skeletal and developmental defects, bone marrow (BM) failure, and a high predisposition to cancer [1, 7, 8]. One of the common clinical features of FA is hematologic manifestations, possibly due to defects in hematopoietic stem cells (HSCs) [9–11]. A majority of FA patients invariably experience progressive BM failure, and oftentimes progress to myelodysplastic syndrome (MDS) and acute myeloid leukemia (AML) [1, 12–14]. Marrow dysfunction, which occurs at an early stage, is associated with HSC loss and accounts for the majority of FA childhood mortality [15–17]. In addition, FA patients are at extremely high risk of developing AML and solid tumors [12, 13].

Upregulation of the tumor suppressor p53 has been shown to play a role in certain hematologic diseases, such as BM failure syndromes and MDS. Specifically, upregulation of p53 function, due to specific genetic lesions in ribosomal biogenesis, leads to apoptosis of erythroid precursors, resulting in pathogenetic features of Diamond–Blackfan anemia (DBA), Schwachman–Bodian–Diamond syndrome (SBDS) and 5q-MDS [18–22]. In FA, it has been reported that p53 deficiency increased cancer development in patients with FA and FA mice [23–26]. Conversely, p53 overactivation caused HSPC depletion in the BM of FA patients [27]. In this study, we demonstrate that the level of p53 protein is critical for the leukemic stem cells from FA patients to repopulate immunodeficient mice.

To examine the functional relevance of *p53* expression in human FA leukemia cells in vivo, we established a FA AML xenotransplant model using primary samples from FA patients with AML and the humanized immunodeficient mouse strain NSGS, which expresses transgenic cDNAs encoding human SCF, GM-CSF, and IL-3 [28]. We first tested the engraftment of three healthy and five AML patient samples with different levels of p53 proteins (Fig. 1a). We found that only two FA patient samples (AML-3 and AML-4) resulted in > 5% human chimeras at 12 weeks post-transplant (Fig. 1b). Additionally, it appeared that AML-3 and AML-4 donor cells underwent myeloid expansion in NSGS recipients (Fig. 1c). We then performed secondary transplantation to examine the effect of p53 on the ability of the FA AML leukemic stem cells (LSCs) to repopulate the NSGS mice. All eight of the secondary recipients of AML-3 cells died of leukemia within 180 days (Fig. 1d). This indicates that the expanded donor AML-3 cells in the primary recipients contained pre-LSCs that induced leukemia in the secondary recipients. While half of the AML-4 secondary recipients (3/6) died of leukemia, three AML-4 secondary recipients survived for 200 days after transplant (Fig. 1d). Interestingly, human-derived BM cells (hCD45^+^) from these three surviving recipients expressed higher levels of p53 than the other three leukemic recipients (Fig. 1e). Thus, the level of p53 protein determines the capacity of the LSCs from FA patients to repopulate immunodeficient mice.

**Fig. 1:**
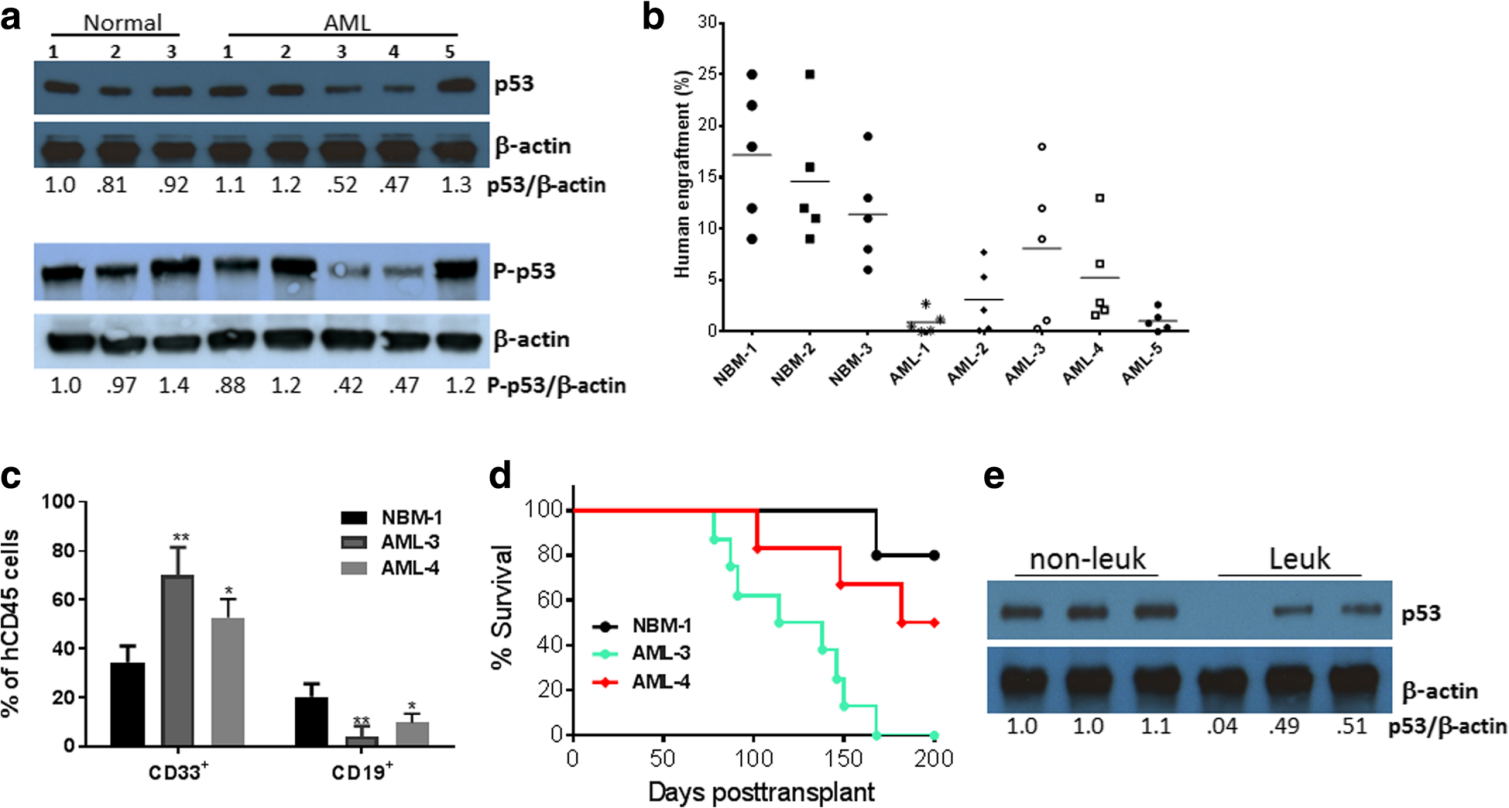
Effect of p53 protein level on human FA leukemia cells. **a** The levels of p53 protein in FA AML cells. BM cells from three healthy donors and five FA AML patients were subjected to immunoblot analysis using antibodies specific for total p53, phosphor-p53 (P-p53), or β-actin. The relative levels of total p53 or of P-p53 to β-actin are indicated below the blot. **b** Comparison of human cells engrafted in the BM of NSGS recipient mice. Mice were transplanted by intra-femoral injection with 1–3 × 10^6^ BM cells from three healthy donors and five FA AML patients. Assessment of xenografts in the BM of the recipient mice was performed 12 weeks after transplantation by BM aspiration and flow cytometry (*n* = 5 per group). **c** BM cells from the recipient mice in **b** were subjected to flow cytometric analysis for human cell contents 12 weeks post-transplant. Quantification of myeloid (CD33^+^) and lymphoid (CD19^+^) cells in total human engraftment (hCD45^+^) (*n* = 5 per group). **d** Survival of transplant recipients. Cells (5 × 10^6^) isolated from the bone marrow of the primary recipients in **b** were injected intrafemorally into each NSGS secondary recipient mouse (*n* = 6–10 for each group). The survival of recipient mice was analyzed with a Kaplan–Meier plot. **e** The levels of p53 protein in human-derived BM cells (hCD45^+^) from three surviving recipients (*non-leuk*) and three leukemic recipients (*Leuk*) transplanted with the AML-4 cells were analyzed by immunoblotting using antibodies for p53 or β-actin. The relative levels of p53 to β-actin are indicated below the blot. The error bars and asterisks in Fig. 1c represent means ± SD and ∗p < 0.05; ∗∗p < 0.01,respectively

The observation that primary FA AML cells with lower levels of p53 induced an earlier onset of leukemia in mice than those with higher p53 levels prompted us to test whether targeted increase of the p53 protein level could ameliorate the FA leukemia burden. Nutlin-3 is a small molecule antagonist of the E3 ubiquitin protein ligase MDM2 that leads to p53 stabilization and has been used in mouse models extensively to reactivate p53 in vivo [29, 30]. We treated the primary recipient mice using a published protocol [31]. Specifically, we treated the mice with Nutlin-3 at a dose of 50 mg/kg daily beginning at 6 weeks post-transplant for 2 weeks, and analyzed the recipients for p53 protein levels and leukemia development in the secondary recipients. We chose primary recipients of AML-3 because this leukemia sample showed low levels of the p53 protein and high xenograft potential (Fig. 2a, b). Nutlin-3 treatment delayed FA leukemia development in the secondary recipient mice, as evidenced by significantly reduced splenomegaly (Fig. 2a) and myeloid expansion (Fig. 2b). We confirmed that Nutlin-3 treatment elevated the p53 protein level in human-derived (hCD45^+^) BM cells of the recipient mice (Fig. 2c). When transplanted into secondary recipient mice, the pre-leukemic cells from the Nutlin-3-treated primary recipient mice showed much less potency in the development of leukemia than vehicle control (Fig. 2d). Thus, increased p53 protein levels by Nutlin-3 treatment ameliorated the FA leukemia burden. However, it should be noted that 60% of the Nutlin-3-treated recipient mice still died of leukemia, suggesting that more factors are at play than just the level of p53 protein.

**Fig. 2:**
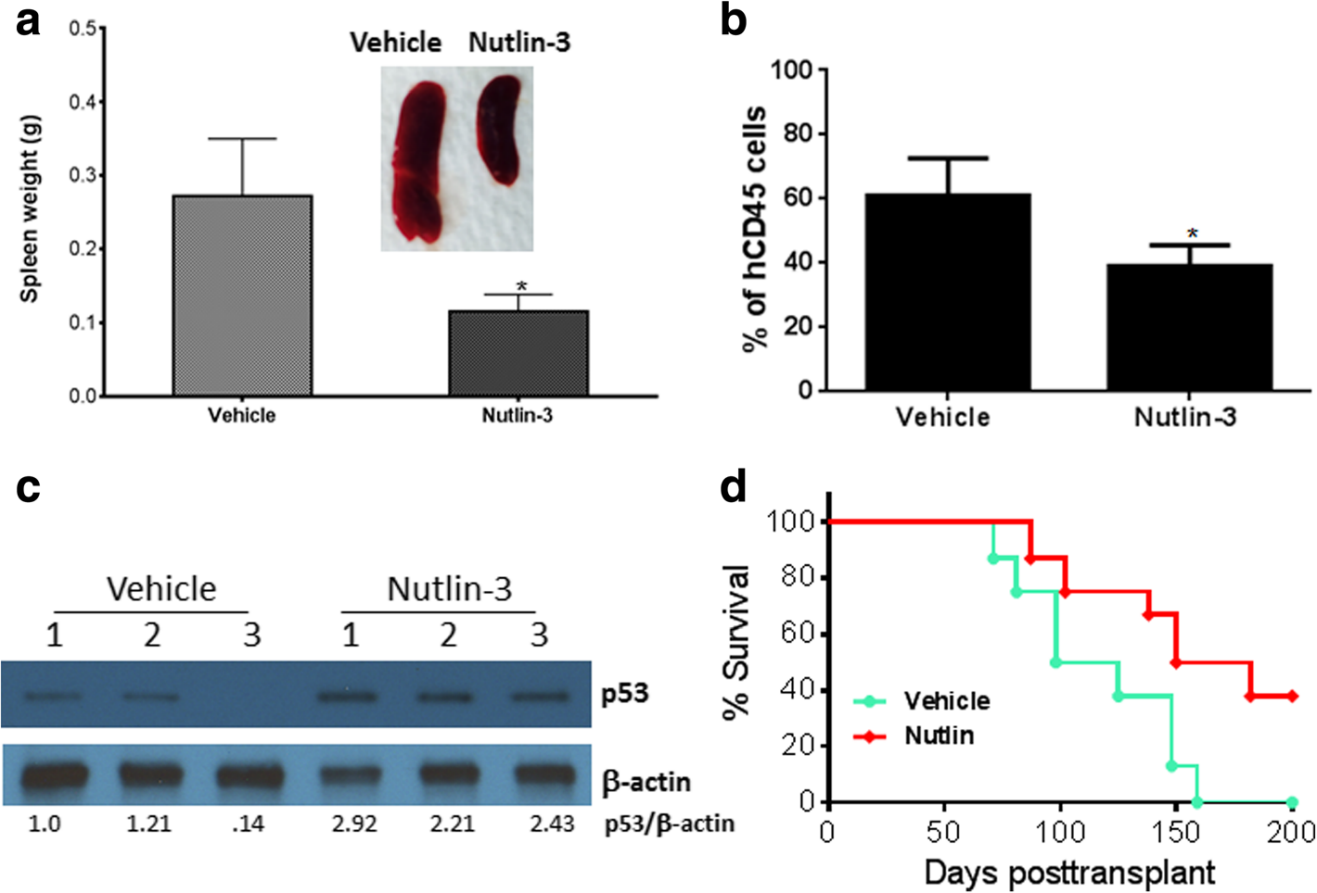
Targeted increase of p53 protein level ameliorates FA leukemia burden. **a** Nutlin-3 treatment ameliorates splenomegaly. We transplanted 1–3 × 10^6^ BM cells from the AML-3 patient by intra-femoral injection into sublethally irradiated NSGS recipient mice. The mice were treated with Nutlin-3 at a dose of 50 mg/kg daily beginning at 6 weeks post-transplant for 2 weeks. Quantification of spleen weights and representative spleen images of the recipient mice are shown (n = 6 per group). **b** Nutlin-3 treatment inhibits myeloid expansion. BM cells from the recipient mice in **a** were subjected to flow cytometric analysis for human cell contents. Quantification of myeloid (CD33^+^) cells in total human engraftment (hCD45^+^) is shown (n = 6 mice per group). **c** Nutlin-3 treatment elevates p53 protein level. The levels p53 protein in human-derived BM cells (hCD45^+^) from three vehicle-treated and three Nutlin-3-treated recipient mice in **a** were analyzed by immunoblotting using antibodies for p53 or β-actin. The relative levels of p53 to β-actin are indicated below the blot. **d** Nutlin-3 treatment delays leukemia development. Cells (5 × 10^6^) isolated from the BM of the primary recipients in **a** were injected intrafemorally into each NSGS secondary recipient m ouse (n = 6–9 for each group). Survival of the recipients was monitored and plotted by Kaplan–Meier curve method. The error bars and asterisks in Fig. 2a, b represent means ± SD and ∗p < 0.05, respectively

In summary, we used primary patient samples to examine the potential link between the status of p53 and FA leukemogenesis. We showed that reduced levels of p53 in FA AML correlate not only with the enhanced ability of the pre-LSCs to repopulate immunodeficient mice but also with increased myeloid expansion and leukemia development. These results are consistent with the well-established roles of p53 in genomic stability, cell cycle arrest, and apoptosis. We also demonstrated that the small-molecule MDM2 inhibitor Nutlin-3, which effectively elevated p53 protein levels in FA AML cells, significantly diminished leukemia burden in our FA AML xenotransplant model. Encouragingly, Nutlin-3 has been used in early clinical trials for cancer indications [32], suggesting that a reactivation-based p53 manipulation approach for FA leukemia could be readily translatable to clinical studies. While our results caution targeting overactive p53 in ameliorating FA HSC loss, restoring p53 activity in FA pre-leukemic HSCs, capable of preventing leukemic transformation, is worthy of investigation as a new avenue for FA leukemia.
